# Toxic epidermal necrolysis, DRESS, AGEP: Do overlap cases exist?

**DOI:** 10.1186/1750-1172-7-72

**Published:** 2012-09-25

**Authors:** Sophie Bouvresse, Laurence Valeyrie-Allanore, Nicolas Ortonne, Marie Pauline Konstantinou, Sylvia H Kardaun, Martine Bagot, Pierre Wolkenstein, Jean-Claude Roujeau

**Affiliations:** 1 Department of Dermatology, Referral center for toxic and auto-immune blistering diseases, Henri Mondor Hospital, Assistance Publique Hôpitaux de Paris Université Paris-Est Créteil, Créteil Cedex, F-94010, France; 2 LIC EA 4393, Henri Mondor Hospital, Assistance Publique Hôpitaux de Paris Université Paris-Est Créteil, Créteil Cedex, F-94010, France; 3 Department of Pathology, Henri Mondor Hospital, Assistance Publique Hôpitaux de Paris, Université Paris-Est Créteil, Créteil Cedex, F-94010, France; 4 Department of Dermatology, University Medical Centre Groningen, University of Groningen, Hanzeplein 1, Groningen, 9713 GZ, The Netherlands; 5 Department of Dermatology, Saint-Louis Hospital, Université Paris VII, Assistance Publique Hôpitaux de Paris, Paris, France

**Keywords:** Toxic epidermal necrolysis, DRESS, AGEP, Severe cutaneous adverse reactions, Overlap

## Abstract

**Background:**

Severe cutaneous adverse reactions to drugs (SCARs) include acute generalized exanthematous pustulosis (AGEP), drug reaction with eosinophilia and systemic symptoms (DRESS) and epidermal necrolysis (Stevens-Johnson syndrome–toxic epidermal necrolysis [SJS-TEN]). Because of the varied initial presentation of such adverse drug reactions, diagnosis may be difficult and suggests overlap among SCARs. Overlapping SCARs are defined as cases fulfilling the criteria for definite or probable diagnosis of at least 2 ADRs according to scoring systems for AGEP, DRESS and SJS-TEN. We aimed to evaluate the prevalence of overlap among SCARs among cases in the referral hospital in France.

**Methods:**

We retrospectively analyzed data for 216 patients hospitalized in the referral centre over 7 years with a discharge diagnosis of AGEP (n = 45), DRESS (n = 47), SJS-TEN (n = 80) or “drug rash” (n = 44). Each case with detailed clinical data and a skin biopsy specimen was scored for AGEP, DRESS and SJS-TEN by use of diagnostic scores elaborated by the RegiSCAR group.

**Results:**

In total, 45 of 216 cases (21%) had at least 2 possible diagnoses: 35 had a single predominant diagnosis (definite or probable), 7 had several possible diagnoses and 3 (2.1% of 145 confirmed SCARs) were overlap SCARs.

**Conclusions:**

Despite ambiguities among SCARs, confirmed overlap cases are rare. This study did not avoid pitfalls linked to its retrospective nature and selection bias. In the acute stage of disease, early identification of severe ADRs can be difficult because of clinical or biologic overlapping features and missing data on histology, biology and evolution. Retrospectively analyzing cases by use of diagnostic algorithms can lead to reliable discrimination among AGEP, DRESS and SJS-TEN.

## Background

Adverse cutaneous reactions to drugs are frequent, affec-ting 2% to 3% of all hospitalized patients [1]. Only about 2% of these adverse cutaneous reactions are considered severe [1]. The spectrum of severe cutaneous adverse reactions to drugs (SCARs) include acute generalized exanthematous pustulosis (AGEP), drug reaction with eosinophilia and systemic symptoms (DRESS) and epidermal necrolysis (Stevens-Johnson syndrome-SJS, toxic epidermal necrolysis -TEN). These conditions are defined by clinical features associated more or less with specific biological and histological findings [2,3].

AGEP is characterized by a pustular eruption arising quickly after administration of the causative drug (usually aminopenicillin, pristinamycin, diltiazem) [4,5]. DRESS, also known as drug-induced hypersensitivity syndrome, is a severe, systemic drug reaction most commonly associated with aromatic anticonvulsants, allopurinol and sulfonamides [6-8]. Patients typically present fever, facial oedema, lymphadenopathy and morbilliform eruption, which may progress to erythematous rash and exfoliative dermatitis. Hematologic abnormalities, including eosinophilia and atypical lymphocytosis, are a hallmark of the condition. Visceral organ involvement typically manifests as hepatitis but may include nephritis, interstitial pneumonitis or myocarditis [8]. Epidermal necrolysis is characterized by extensive epidermal loss with mucous membrane erosions and often presents as impaired general condition. These SCARs are defined as SJS, “transitional SJS-TEN” or TEN, depending on the extent of epidermal detachment (< 10%, 10–30%, > 30%, respectively) [9]. The conditions have been strongly associated with anti-infective sulfonamides, allopurinol, carbamazepine, phenobarbital, phenytoin, oxicam, and more recently nevirapine, lamotrigine and amifostine [10,11].

For each of these SCARs, diagnostic criteria have been established [4,8]. These scoring systems take into account clinical patterns (presentation, evolution), biological data (for AGEP and DRESS) and histological findings. The diagnostic scales are used to retrospectively score data and with consensus to classify cases as definitive, probable, possible or excluded.

Because the initial presentation of such adverse drug reactions may vary, diagnosis is difficult and suggests the possibility of overlap among SCARs. For instance, cases of AGEP may present facial oedema, atypical targets or blisters [12,13], and 20% of cases may show mucous involvement [4]. Early descriptions of AGEP pointed to non-rare suspicion of TEN with a confluence of pustules resulting in superficial detachment, and even recently AGEP cases similar to TEN were reported [14]. Elevated neutrophil count may be accompanied by mild eosinophilia in up to one-third of cases in certain series [15]. Internal organ involvement is not common in AGEP, although lymph-node enlargement [15], slightly reduced creatinine clearance or slight elevation of liver enzyme levels may be observed [4].

Concerning DRESS, pustules may be found in up to 20% of cases [16]. Vesicles, blisters, atypical target lesions or mucous membrane involvement have been reported [6], occasionally with mild mucosal erosions [17]. Cases of “overlap” between DRESS and TEN have been reported, which suggests the difficulty in classifying these SCARs under certain circumstances [18].

Finally, in SJS-TEN, internal organ involvement is not rare and can include elevated levels of liver enzymes, eosinophilia, and transitory proteinuria [19-21].

Therefore, because several conditions are suspected, clinicians may have difficulty diagnosing these SCARs [22]. Cases of overlapping SCARs – fulfilling diagnostic criteria for different SCARs -- may exist. We investigated cases of SCARs in our referral center to determine prevalence of overlapping SCARs, defined as fulfilling the criteria for definite or probable diagnosis of at least two ADRs accor-ding to scoring systems for AGEP, DRESS and SJS-TEN.

## Patients and methods

### Selection of cases

We conducted a retrospective monocentric study of all patients hospitalised in our department between January 1, 2000 and December 31, 2006 with a discharge diagnosis of AGEP, DRESS, SJS-TEN or “drug rash not otherwise specified” and with an available skin biopsy. Exclusion criteria were missing data concerning clinical presentation or biological results and wrong discharge diagnosis.

We collected clinical and epidemiological data on demographic characteristics, exposure to drugs, clinical presentation of the SCAR (maximal body temperature, lymph node enlargement, duration and description of the rash, mucosal involvement, presence of erosions and extent), biological data (e.g., leucocyte counts, hepatic and renal function, serological data, blood cultures) and pathology results of skin biopsy.

### Classification of cases

Three of us (JCR, LVA, SB) used predefined RegiSCAR algorithms [4,8] to assess drug causality with clinical data from the patient’s file or clinical pictures, as well as pathology reports, with blinding to exposure to risk factors, patient identity and biological data.

Each case was scored for AGEP, DRESS and SJS-TEN and classified as definitive, probable, or possible AGEP, DRESS or SJS-TEN or excluded. Overlap SCARs were defined as cases fulfilling the criteria for definite or probable diagnosis of at least 2 ADRs according to the scoring systems. Data were entered into Excel spreadsheets and checked for entry-related errors.

## Results

**Figure 1:**
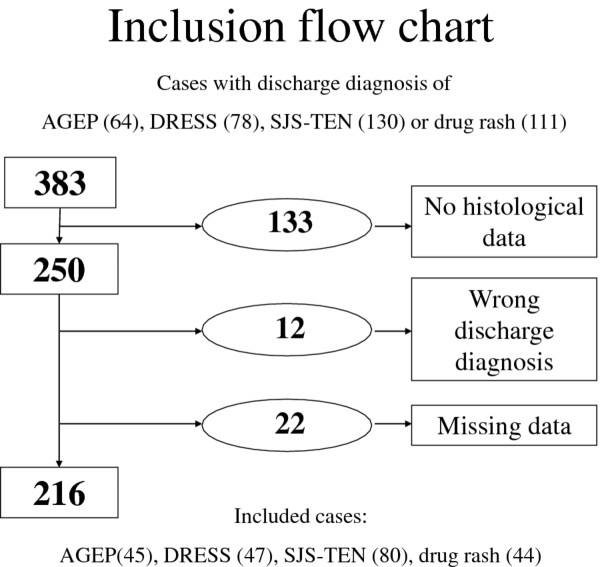
Flow chart of selection of cases in the study.

Among 383 patient cases with a discharge diagnosis of AGEP, DRESS, SJS-TEN or “drug rash”, only 250 were selected because a skin biopsy specimen was available. Data for 34 cases were excluded because of missing data for clinical presentation or biological results or wrong discharge diagnosis (Table 1). Eventually, 216 cases were analysed: 45 cases with a discharge diagnosis of AGEP, 47 with DRESS, 80 with SJS-TEN and 44 with drug rashes. The flow chart of the selection of cases is in Figure 1.

**Table 1:** Details of missing data by adverse reactions

		**AGEP**	**DRESS**	**SJS-TEN**	**Drug rash**	**Total**
	**Number of cases**	**64**	**78**	**111**	**130**	**383**
**Excluded cases**	**Missing data: - clinical or biological- file**	**2**	**2**	**3**	**1**	**8**
		**1**	**1**	**-**	**12**	**14**
	**Wrong discharge diagnosis**	**1**	**1**	**8**	**2**	**12**
	**Total**	**4**	**4**	**11**	**15**	**34**
**Missing histology**		**15**	**27**	**35**	**56**	**133**
**Cases included**		**45**	**47**	**80**	**44**	**216**

**Figure 2:**
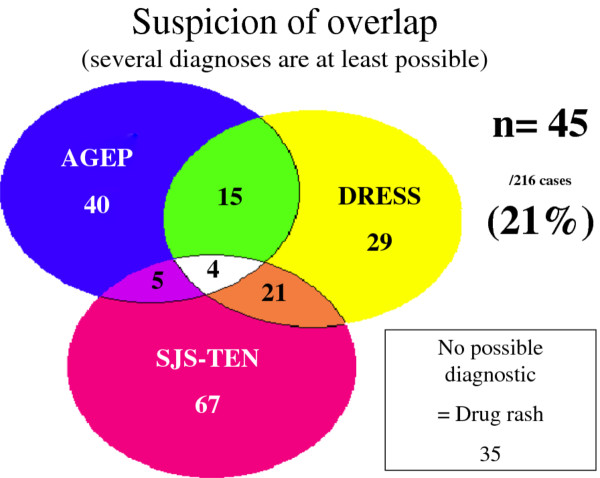
Results of the validation : several diagnosis are at least possible.

**Figure 3:**
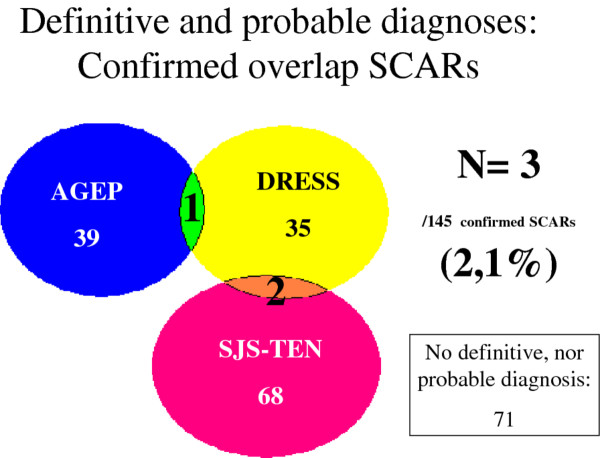
Results of the validation including only probable and definite diagnosis.

According to the scoring systems, among these 216 cases, 181 cases had at least one possible diagnosis: 107 had only one diagnosis (with definite or probable disease), 35 had 3 excluded diagnoses, 29 had a possible single diagnosis. The remaining 45 (21%) had several possible, distinct SCARs (possible, probable or definite) (Figure 2). Among the 45 cases, 35 had a single predominant diagnosis (definite or probable), 7 had several possible diagnoses and 3 (2.1% of confirmed 145 confirmed SCARs cases) were “true” overlap, with definite or probable diagnosis of 2 distinct SCARs: one overlap between AGEP and DRESS and 2 overlaps between SJS-TEN and DRESS (Figure 3).

## Discussion

Because the initial presentation of severe adverse drug reactions may vary, diagnosis may be difficult in that several conditions may be suspected. Our retrospective study of cases of SCARs in our referral centre revealed the frequent occurrence (n = 45; 21%) of SCARs cases of several possible diagnoses (possible, probable or certain), which reflects the clinical ambiguity among several SCARs. In such situations, the clinician is confronted with an uncertain diagnosis of several disease entities. For these 45 cases, when several diagnoses were at least possible, the retrospective assignment of a score often led to a single final diagnosis. Indeed, for 35 of the 45 cases, we could establish a single predominant diagnosis (probable or definite), with one (or several) other possible diagnosis(es). Seven of the 10 remaining cases could not be classified because several SCARs were possible. Only 3 “true” overlap SCARs were documented, representing 2.1% of the 145 confirmed cases of SCARs.

Finally, for the patient with a severe cutaneous reaction, determining the offending drug may be more important than a precise diagnosis with the reaction but is not totally acceptable because of different risks and the nature of long-term sequelae. Sequelae have never been reported after AGEP, are infrequent after DRESS and mostly auto-immune [23], and are nearly always present after SJS/TEN [24]. Therefore, follow-up will differ depending on the final diagnosis.

Not all SCARs have a similar weight in the evaluation of the benefit/risk balance by regulatory agencies and pharmaceutical companies. The mortality rate is 10 times lower for AGEP (2%) than SJS/TEN (20–25%) [10]. For a newly released drug, the report of one case as “AGEP” will have a different impact than a report of “TEN”.

The pathophysiology of drug eruption is not completely clear. AGEP, DRESS and SJS-TEN are all categorized as type IV reactions according to the classification by Coombs and Gell. Type IV reactions are mediated by T cells, causing so-called delayed hypersensitivity [25,26]. Recently, several immunohistochemical and functional studies of drug-reactive T cells in patients with distinct forms of SCARs revealed that distinct T-cell functions led to different clinical phenotypes. These T cells recruit and activate monocytes, eosinophils or neutrophils. Drug-specific T cells also orchestrate inflammatory skin reactions by releasing various cytokines and chemokines. For instance, granulysin is the dominant cytokine inducing the destruction of epidermis in SJS-TEN [27], eotaxine and IL5 in DRESS [28,29], and IL-8, IL-17 and IL-22 in AGEP [30,31]. In considering the heterogeneity of T-cell function, Pichler *et al.* suggested further sub-classifying delayed hypersensitivity reactions into T-cell reactions, which through the release of certain cytokines and chemokines preferentially activate and recruit monocytes (type IVa), eosinophils (type IVb), or neutrophils (type IVD) [32]. Various drug-hypersensitivity diseases can be related to the preferential activation of drug-specific T cells with distinct functions. These complexe immune reactions are not exclusive and may be combined. An overlap of immune reactions is common, even if one type is often dominant, and would explain clinical ambiguities among SCARs [32,33]. Research, especially into immunological mechanisms and pharmacogenetics, should be based on well-characterized phenotypes and drug causality. In such settings, diagnostic ambiguity could skew the results and must be avoided. A striking example was the finding in Taiwan that among carbamazepine-related SCARs, human leukokyte antigen B*1502 positivity was associated with all SJS or TEN cases but no cases of DRESS or mild eruptions [34]. If investigations had been performed on all carbamazepine-related cutaneous reactions, such clear results would not be missed.

The limitations of this study include its retrospective nature and selection bias. Indeed, patients hospitalized for SCARs may show a more severe or atypical presentation. The study was monocentric, and our department is a referral center for toxic bullous diseases, so recruitment of patients may have been biased because cases are addressed when particularly life-threatening. Cases were selected by exhaustivity of discharge diagnosis charts and their validity. Missing data might have caused the loss of many cases and could contribute to underestimating overlap cases. Interpretation bias was limited because clinical and histological data were scored by use of a predefined scoring algorithm, and the review committee was blinded to patient identity and exposure to risk factors and biological data. The strengths of this retrospective study are its exhaustiveness and use of pre-established diagnostic scales.

## Conclusions

Our results suggest that even if ambiguities among SCARs are not rare, confirmation of overlap cases are rare. AGEP, DRESS and SJS-TEN are distinct entities with no evidence of a wide pathological spectrum. Differentiating different SCARs may lead to quicker diagnosis and more effective disease management.

## Competing interest

The authors declare that they have no competing interests.

## Authors’ contributions

SB, LVA, JCR have made substantial intellectual contributions to conception and design. SB, LVA, JCR, MPK and NO have made substantial contributions to acquisition of data, or analysis and interpretation of data. SB, LVA, JCR, NO, MB and PW have been involved in drafting the manuscript or revising it critically for important intellectual content. All the authors have given final approval of the version submitted for publication.

## Authors’ information

This work was presented in part as an oral communication at Journées Dermatologiques de Paris, France in December 2007 and at the 3^rd^ Drug Hypersensitivity Meeting, Paris 2008 as a poster presentation.

## References

[B1] Wolf R Orion E Marcos B Matz H Life-threatening acute adverse cutaneous drug reactions Clin Dermatol 2005 23 2 171 181 15802211 10.1016/j.clindermatol.2004.06.012

[B2] Roujeau JC Stern RS Severe adverse cutaneous reactions to drugs N Engl J Med 1994 331 19 1272 1285 10.1056/NEJM199411103311906 7794310

[B3] Valeyrie-Allanore L Sassolas B Roujeau JC Drug-induced skin, nail and hair disorders Drug Saf 2007 30 11 1011 1030 10.2165/00002018-200730110-00003 17973540

[B4] Sidoroff A Halevy S Bavinck JN Vaillant L Roujeau JC Acute generalized exanthematous pustulosis (AGEP)-a clinical reaction pattern J Cutan Pathol 2001 28 3 113 119 10.1034/j.1600-0560.2001.028003113.x 11168761

[B5] Sidoroff A Dunant A Viboud C Risk factors for acute generalized exanthematous pustulosis (AGEP) – results of a multinational case–control study (EuroSCAR) Br J Dermatol 2007 157 5 989 996 10.1111/j.1365-2133.2007.08156.x 17854366

[B6] Bocquet H Bagot M Roujeau JC Drug-induced pseudolymphoma and drug hypersensitivity syndrome (Drug Rash with Eosinophilia and Systemic Symptoms: DRESS) Semin Cutan Med Surg 1996 15 4 250 257 10.1016/S1085-5629(96)80038-1 9069593

[B7] Cacoub P Musette P Descamps V The DRESS syndrome: a literature review Am J Med 2011 124 7 588 597 10.1016/j.amjmed.2011.01.017 21592453

[B8] Kardaun SH Sidoroff A Valeyrie-Allanore L Halevy S Davidovici BB Mockenhaupt M Roujeau JC Variability in the clinical pattern of cutaneous side-effects of drugs with systemic symptoms: does a DRESS syndrome really exist? Br J Dermatol 2007 156 3 609 611 10.1111/j.1365-2133.2006.07704.x 17300272

[B9] Auquier-Dunant A Mockenhaupt M Naldi L Correia O Schröder W Roujeau JC SCAR Study Group. Severe Cutaneous Adverse Reactions Correlations between clinical patterns and causes of erythema multiforme majus, Stevens-Johnson syndrome, and toxic epidermal necrolysis: results of an international prospective study Arch Dermatol 2002 138 8 1019 1024 10.1001/archderm.138.8.1019 12164739

[B10] Mockenhaupt M Viboud C Dunant A Naldi L Halevy S Bouwes Bavinck JN Sidoroff A Schneck J Roujeau JC Flahault A Stevens-Johnson syndrome and toxic epidermal necrolysis: assessment of medication risks with emphasis on recently marketed drugs. The EuroSCAR-study J Invest Dermatol 2008 128 1 35 44 10.1038/sj.jid.5701033 17805350

[B11] Valeyrie-Allanore L Poulalhon N Fagot JP Sekula P Davidovici B Sidoroff A Mockenhaupt M Stevens-Johnson syndrome and toxic epidermal necrolysis induced by amifostine during head and neck radiotherapy Radiother Oncol 2008 87 2 300 303 10.1016/j.radonc.2008.01.021 18328585

[B12] Roujeau JC Bioulac-Sage P Bourseau C Guillaume JC Bernard P Lok C Plantin P Claudy A Delavierre C Vaillant L Wechsler J Danan G Bénichou C Beylot C Acute generalized exanthematous pustulosis. Analysis of 63 cases Arch Dermatol 1991 127 9 1333 1338 10.1001/archderm.1991.01680080069004 1832534

[B13] Speeckaert MM Speeckaert R Lambert J Brochez L Acute generalized exanthematous pustulosis: an overview of the clinical, immunological and diagnostic concepts Eur J Dermatol 2010 20 4 425 433 20542841 10.1684/ejd.2010.0932

[B14] Peermohamed S Haber RM Acute generalized exanthematous pustulosis simulating toxic epidermal necrolysis: a case report and review of the literature Arch Dermatol 2011 147 6 697 701 10.1001/archdermatol.2011.147 21690532

[B15] Machet ML Vaillant L Acute generalized exanthematic pustulosis Ann Dermatol Venereol 2001 128 1 73 79 11226910

[B16] Kleier RS Breneman DL Boiko S Generalized pustulation as a manifestation of the anticonvulsant hypersensitivity syndrome Arch Dermatol 1991 127 9 1361 1364 10.1001/archderm.1991.01680080097009 1832535

[B17] Begon E Roujeau JC Drug hypersensitivity syndrome: DRESS (Drug Reaction with Eosinophilia and Systemic Symptoms) Ann Dermatol Venereol 2004 131 3 293 297 10.1016/S0151-9638(04)93600-9 15107755

[B18] Wolf R Davidovici B Matz H Mahlab K Orion E Sthoeger ZM Drug Rash with eosinophilia and systemic symptoms versus Stevens-Johnson Syndrome–a case that indicates a stumbling block in the current classification Int Arch Allergy Immunol 2006 141 3 308 310 10.1159/000095437 16940741

[B19] Bombal C Roujeau JC Kuentz M Revuz J Touraine R Hematologic anomalies in Lyell’s syndrome. Study of 26 cases Ann Dermatol Venereol 1983 110 2 113 119 6881853

[B20] Lebargy F Wolkenstein P Gisselbrecht M Lange F Fleury-Feith J Delclaux C Roupie E Revuz J Roujeau JC Pulmonary complications in toxic epidermal necrolysis: a prospective clinical study Intensive Care Med 1997 23 12 1237 1244 10.1007/s001340050492 9470079 PMC7095164

[B21] Blum L Chosidow O Rostoker G Philippon C Revuz J Roujeau JC Renal involvement in toxic epidermal necrolysis J Am Acad Dermatol 1996 34 6 1088 1090 10.1016/S0190-9622(96)90297-2 8647982

[B22] Valeyrie-Allanore L Roujeau JC Denominations and classification of severe cutaneous adverse reactions to drugs: splitters versus mergers Eur J Dermatol 2007 17 5 359 360 17673376 10.1684/ejd.2007.0230

[B23] Funck-Brentano E Duong T Family D Bouaziz JD Ortonne N Bagot M Roujeau JC Wolkenstein P Valeyrie-Allanore L Auto-immune thyroiditis and drug reaction with eosinophilia and systemic symptoms (DRESS) associated with HHV-6 viral reactivation Ann Dermatol Venereol 2011 138 8–9 580 585 21893231 10.1016/j.annder.2011.01.048

[B24] Geudry J Roujeau JC Binaghi M Soubrane G Muraine M Risk factors for the development of ocular complications of Stevens–Johnson syndrome and toxic epidermal necrolysis Arch Dermatol 2009 145 2 157 162 10.1001/archdermatol.2009.540 19221260

[B25] Pichler WJ Naisbitt DJ Park BK Immune pathomechanism of drug hypersensitivity reactions J Allergy Clin Immunol 2011 127 3 S74 S81 10.1016/j.jaci.2010.11.048 21354503

[B26] Pichler WJ Adam J Daubner B Gentinetta T Keller M Yerly D Drug hypersensitivity reactions: pathomechanism and clinical symptoms Med Clin North Am 2010 94 4 645 664 10.1016/j.mcna.2010.04.003 20609855

[B27] Chung WH Hung SI Chen YT Genetic predisposition of life-threatening antiepileptic-induced skin reactions Expert Opin Drug Saf 2010 9 1 15 21 10.1517/14740330903427969 20001755

[B28] Picard D Janela B Descamps V D’Incan M Courville P Jacquot S Rogez S Mardivirin L Moins-Teisserenc H Toubert A Benichou J Joly P Musette P Drug reaction with eosinophilia and systemic symptoms (DRESS): a multiorgan antiviral T cell response Sci Transl Med 2010 2 46 46ra62 10.1126/scitranslmed.3001116 20739682

[B29] Yawalkar N Shrikhande M Hari Y Nievergelt H Braathen LR Pichler WJ Evidence for a role for IL-5 and eotaxin in activating and recruiting eosinophils in drug-induced cutaneous eruptions J Allergy Clin Immunol 2000 106 6 1171 1176 10.1067/mai.2000.110922 11112902

[B30] Britschgi M Steiner UC Schmid S Depta JP Senti G Bircher A Burkhart C Yawalkar N Pichler WJ T-cell involvement in drug-induced acute generalized exanthematous pustulosis J Clin Invest 2001 107 11 1433 1441 10.1172/JCI12118 11390425 PMC209321

[B31] Sugita K Kabashima K Sawada Y Haruyama S Yoshioka M Mori T Kobayashi M Ogasawara K Tokura Y Increased circulating Th17 frequencies and serum IL-22 levels in patients with acute generalized exanthematous pustulosis J Eur Acad Dermatol Venereol 2011 25 4 485 488 10.1111/j.1468-3083.2010.03771.x 20569282

[B32] Pichler WJ Delayed drug hypersensitivity reactions Ann Intern Med 2003 139 8 683 693 14568857 10.7326/0003-4819-139-8-200310210-00012

[B33] Roujeau JC Clinical heterogeneity of drug hypersensitivity Toxicology 2005 209 2 123 129 10.1016/j.tox.2004.12.022 15767024

[B34] Hung SI Chung WH Jee SH Chen WC Chang YT Lee WR Hu SL Wu MT Chen GS Wong TW Hsiao PF Chen WH Shih HY Fang WH Wei CY Lou YH Huang YL Lin JJ Chen YT Genetic susceptibility to carbamazepine-induced cutaneous adverse drug reactions Pharmacogenet Genomics 2006 16 4 297 306 10.1097/01.fpc.0000199500.46842.4a 16538176

